# Efficacy of a reading and language intervention for children with Down syndrome: a randomized controlled trial

**DOI:** 10.1111/j.1469-7610.2012.02557.x

**Published:** 2012-04-26

**Authors:** Kelly Burgoyne, Fiona J Duff, Paula J Clarke, Sue Buckley, Margaret J Snowling, Charles Hulme

**Affiliations:** 1 Down Syndrome Education InternationalPortsmouth, UK; 2 Centre for Reading and Language, University of YorkUK; 3 School of Education, University of LeedsUK; 4 Division of Psychology and Language Sciences, University College LondonUK

**Keywords:** Down syndrome, early literacy, intervention, language, phonological awareness, RCT

## Abstract

**Background:**

This study evaluates the effects of a language and literacy intervention for children with Down syndrome.

**Methods:**

Teaching assistants (TAs) were trained to deliver a reading and language intervention to children in individual daily 40-min sessions. We used a waiting list control design, in which half the sample received the intervention immediately, whereas the remaining children received the treatment after a 20-week delay. Fifty-seven children with Down syndrome in mainstream primary schools in two UK locations (Yorkshire and Hampshire) were randomly allocated to intervention (40 weeks of intervention) and waiting control (20 weeks of intervention) groups. Assessments were conducted at three time points: pre-intervention, after 20 weeks of intervention, and after 40 weeks of intervention.

**Results:**

After 20 weeks of intervention, the intervention group showed significantly greater progress than the waiting control group on measures of single word reading, letter-sound knowledge, phoneme blending and taught expressive vocabulary. Effects did not transfer to other skills (nonword reading, spelling, standardised expressive and receptive vocabulary, expressive information and grammar). After 40 weeks of intervention, the intervention group remained numerically ahead of the control group on most key outcome measures; but these differences were not significant. Children who were younger, attended more intervention sessions, and had better initial receptive language skills made greater progress during the course of the intervention.

**Conclusions:**

A TA-delivered intervention produced improvements in the reading and language skills of children with Down syndrome. Gains were largest in skills directly taught with little evidence of generalization to skills not directly taught in the intervention.

## Introduction

Down syndrome (DS) is the most common genetic cause of learning disability and is associated with particular difficulties with language and communication. Despite this, most individuals with DS can learn to read, though attainment levels vary widely (Byrne, MacDonald, & Buckley, 2002; Laws & Gunn, 2002). There remains, however, limited evidence about how best to intervene to improve these children’s reading and language skills. Although a great deal of evidence supports the use of phonics for the teaching of reading (DCSF, 2009; National Reading Panel, 2000; Rose, 2006), there is debate about the appropriateness of this approach for children with DS. Typically, children with DS show good visual skills (Fidler, Most, & Guiberson, 2005), deficits in phonological awareness (Cossu, Rossini, & Marshall, 1993; Lemons & Fuchs, 2010a) and a profile of stronger word recognition than decoding skills. This profile has led some to advocate the use of ‘whole-word’ strategies for teaching these children to read. Within the ‘Triangle Model’ of reading (Seidenberg & McClelland, 1989), such an approach might be seen as fostering the use of the ‘semantic’ pathway linking orthography with word meanings. Although this can undoubtedly increase the number of words children recognise, it does little to foster the development of a ‘phonological’ pathway mapping orthography to phonology, which is fundamental to independent reading.

A small number of studies have demonstrated that children with DS benefit from reading instruction which targets phonological awareness and reading skills (e.g. Cologon, Cupples, & Wyver, 2011; Goetz et al., 2008; Lemons & Fuchs, 2010b). However, the available evidence is limited by small samples (*N* = 7–24), short training periods (10 hr to 16 weeks of daily 40-min intervention), a lack of appropriate comparison groups, and inclusion criteria which do not include the full range of abilities seen in individuals with DS. Furthermore, little is known about individual differences in response to intervention.

Among typically developing children, one of the main factors which affects response to intervention is oral language skill (Vadasy, Sanders, & Abbott, 2008; Whiteley, Smith, & Connors, 2007). Other child-variables which affect response include (in order of effect size): rapid naming, behaviour, phonological awareness, alphabetic knowledge, memory, IQ and demographics (Nelson, Benner, & Gonzalez, 2003) and extrinsic factors such as the length and quality of instruction are also important (Al Otaiba & Fuchs, 2002; National Reading Panel, 2000).

With these findings as a backdrop, we set out to evaluate a programme of intervention for children with DS which combined phonics-based reading instruction with vocabulary teaching. The rationale for the intervention was that language impairments are common in children with DS (e.g. Abbeduto, Warren, & Conners, 2007) and if attenton is not paid to these they may compromise the development of phoneme awareness (Carroll, Snowling, Stevenson, & Hulme, 2003; Metsala & Walley, 1998). Moreover, a previous study had shown that such an approach was effective for supporting the reading development of typically developing children who show poor response to intervention (Duff et al., 2008). Thus, we report a randomized controlled trial (RCT) of an intervention for children with DS that targets both reading and language skills. The size and scope of our study enables us to investigate whether the intervention accelerates progress in reading and language when compared with ‘teaching as usual’ and the factors that predict response to intervention.

## Method

**Figure 1:**
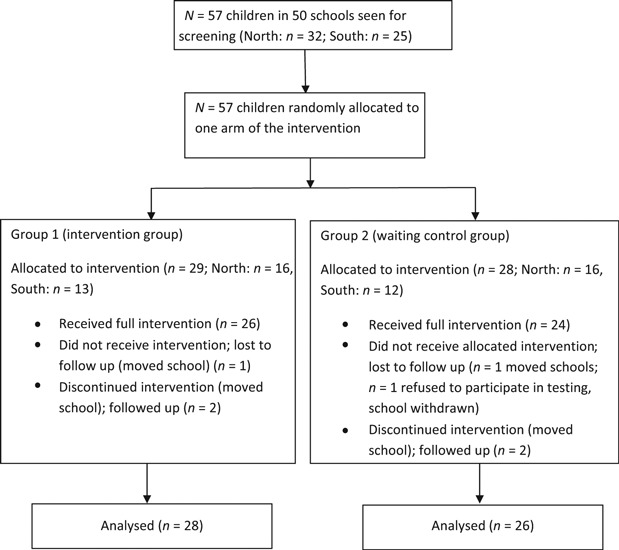
Flow diagram showing participant recruitment and progress through the trial [in line with CONSORT recommendations (Schulz, Altman, & Moher, 2010)]

Trained TAs delivered a reading and language intervention to children on an individual basis in daily 40-min sessions in the children’s schools. The performance of children who received the intervention for 40 weeks (intervention group) was compared to a waiting control group who continued with their regular education during the first 20 weeks (which included one-to-one support from a TA) before receiving the intervention in just the second 20-week period. Ethical approval was granted by the Ethics Committee, Department of Psychology, University of York; informed parental consent was obtained for all children. The trial was conducted within schools and hence was not registered. Details of participant recruitment, allocation and flow through the study are summarised in the CONSORT diagram (Figure 1).

### Participants

The project was advertised in Spring 2009 to parents and educators of children with DS attending primary schools in two UK locations: North (Yorkshire) and South (Hampshire). Fifty-eight children in 50 schools were identified; all children were in integrated classrooms with support from a TA for a large part of the school day. Fifty-seven children (one child was unavailable for testing) were visited in school for an initial assessment. The only eligibility criterion was that children should be in primary school Years 1–5 at the start of the study so that they would remain in primary education for the duration of the project. The 57 children (28 boys) recruited were randomly allocated to either the intervention or waiting control group.

Children were aged between 5:02 and 10:00 at the start of the project. Children came from a range of SES backgrounds but were predominantly from white, English-speaking families; two children spoke an additional language (Cantonese, Russian). Parent-completed questionnaires (84% returned) indicated high rates of involvement in early services [portage (*N* = 40), occupational therapy (*N* = 21) and speech and language services (*N* = 42)] though the timing, frequency and duration of service involvement varied widely. Visual impairments were reported for 37 children; 23 children were reported as having a hearing impairment ranging from mild to severe loss in one or both ears. Most parents (96%) reported that they had read to their child regularly from an early age and had tried to teach their child to read.

Teacher ratings on the Strengths and Difficulties Questionnaire (SDQ; Goodman, 1997) (82% returned) indicated significant behavioural problems (i.e. Total difficulties score 16–40) for five children in the intervention group and seven children in the waiting control group. Descriptive statistics for the two groups of children are shown in Table 1.

**Table 1:** Mean raw scores (*SD*) for the intervention and waiting control groups on screening and descriptive measures, prior to the intervention

		Intervention group					
		
		Intervention			Waiting control		
			
Measure (maximum score)	Test point	*N*	*M* (*SD*)	Range	*N*	*M* (*SD*)	Range
Age (months)	Screening	29	80.48 (14.74)	60–115	28	77.82 (15.88)	57–115
SDQ (40)	Screening	27	11.37 (4.66)	3–23	20	13.05 (5.87)	5–24
Single-word reading (30)	Screening	29	4.79 (8.30)	0–29	28	4.50 (7.88)	0–30
Letter-sound knowledge (32)	Screening	29	17.24 (9.84)	0–31	28	14.43 (9.41)	0–28
Expressive vocabulary (170)	Screening	29	29.97 (11.85)	6–63	28	28.79 (13.41)	6–73
Receptive vocabulary (170)	Screening	29	36.93 (12.42)	6–70	28	32.43 (13.84)	5–62
Non-verbal IQ: Block Design (40)	*t*1	28	13.39 (5.83)	0–22	26	11.73 (6.70)	0–24
Non-verbal IQ: object assembly (37)	*t*1	28	10.25 (6.62)	0–24	26	8.65 (6.97)	0–25
Receptive grammar (32)	*t*1	28	12.36 (4.53)	3–22	26	12.50 (3.82)	5–23
Basic concepts (18)	*t*1	28	8.93 (4.74)	0–17	26	9.38 (3.99)	1–18

SDQ, Strengths and Difficulties Questionnaire.

*t*1 is the testing time point immediately prior to the first 20-week block (of intervention or waiting).

### Assessments

Children were assessed four times: at screening, immediately before intervention (*t*1), after the first 20-week intervention period (*t*2), and after the second 20-week intervention period (*t*3). Children were assessed individually over two or more sessions on separate days. TAs were present during testing to assist with behavioural and communicative challenges where necessary. In addition, tasks were kept short, fast-paced and varied to maintain motivation and interest. Here, we only report data for outcomes and predictors relevant to this report.

*Nonverbal IQ (t*1*):* Assessed using Block Design and Object Assembly subtests (WPPSI-III; Wechsler, 2002; alphas 0.84 and 0.85, respectively).

### Reading-related measures

*Single-word reading (screening; t1*–*t3).* All children completed the Early Word Recognition (EWR) test (α = 0.98) from the York Assessment of Reading (YARC) Early Reading battery (Hulme et al., 2009). Children reading over 25 words were given an additional set of words from the Test of Single-Word Reading, from the YARC.

*Letter-sound knowledge (screening; t*1–*t*3*)*: This extended test of alphabetic knowledge from the YARC (Hulme et al., 2009) asks the child to provide the sound for 32 individual letters and digraphs (α = 0.98).

*Phoneme blending (t*1–*t*3*)*: The child was asked to select which of three pictures represented a word spoken by the experimenter in ‘robot’ talk. On each trial a target picture (e.g. *bed*) was presented along with pictures representing an initial phoneme distracter (e.g. *bud*) and a rhyming distracter (e.g. *head*). All targets and distracters had a consonant-vowel-consonant structure. Two practice items were followed by 10 test items (α = 0.67).

*Nonword reading (t*1–*t*3*)*: Children were asked to read the names of six cartoon monsters: ‘et’, ‘om’, ‘ip’, ‘neg’, ‘sab’ and ‘hic’. This test was devised because all available nonword reading tests were too difficult. Two practice items were given before test items (internal reliability = 0.88).

*Spelling (t*1–*t*3*)*: Ten words were presented as pictures to be named and spelled (see also Bowyer-Crane et al., 2008). If no letters were correctly represented in the first two items the test was discontinued (internal reliability = 0.97).

### Language measures

*Vocabulary (t*1–*t*3*)*. Children were given Expressive and Receptive One-Word Picture Vocabulary Tests (EOWPVT; ROWPVT; Brownell, 2000). Median internal consistencies across the relevant age ranges were 0.96.

*Taught vocabulary knowledge (t*1–*t*3*):* Tests were created to measure expressive and receptive knowledge of words explicitly taught in each phase of the intervention programme (i.e. weeks 1–20, tested t1–*t*3; weeks 21–40, tested *t*2–*t*3). Six words of each type (nouns, adverbs, adjectives, prepositions) were tested. In the expressive test, children were shown pictures that they were asked to: name (nouns); say what the person was doing (verbs; e.g. ‘what is the man doing?’‘*Stretching*’); name after a prompt related to a comparison picture (adjectives; e.g. ‘this boy is clean, this boy is...?’‘*Dirty’*); or answer a specific question designed to elicit a preposition (e.g. ‘where is the book?’‘*On* the table’). In the receptive test, children were asked to select the picture (from a choice of 4) which represented the target word. Correlations between standardised and bespoke vocabulary tests ranged from 0.64 to 0.81 (*p*s < .001).

*Expressive grammar and information (t*1–*t*3*)*: Assessed using the Action Picture Test (APT; Renfrew, 1997).

*Basic concept knowledge (t*1*)*: From the Clinical Evaluation of Language Fundamentals (CELF) Preschool 2nd Edition (Wiig, Secord, & Semel, 2006) assessed knowledge of 18 basic linguistic concepts (internal consistency = 0.85).

*Receptive grammar (t*1*)*: Measured by the Test for Reception of Grammar 2 (TROG-2; Bishop, 2003). Eight grammatical constructs were tested in blocks of four items; each correct item was awarded a score of 1 (internal consistency = 0.87).

*Behaviour*: Assessed at *t*1–*t*3 by ratings of video-recordings of assessment sessions. Using a time-sampling technique, behaviour was rated over 10 s periods on a 5-point scale (1 = very good; 5 = very challenging) every 5 min through 60 min of film; scores were averaged to create a single score for each child at each time point (inter-rater reliability = 0.87).

### Outcome measures

Primary outcomes were those proximal to the content of the intervention (letter-sound knowledge, phoneme blending, single word reading, taught vocabulary); secondary outcome measures were those more distal to the content of the intervention (nonword reading, phonetic spelling, standardised tests of receptive and expressive vocabulary and expressive grammar and information).

### Intervention programme

The intervention programme consisted of two components: a Reading Strand and a Language Strand. Four sessions each week were dedicated to new teaching; the fifth session provided an opportunity to revise and consolidate learning. The intervention followed a prescribed programme in daily 40 min sessions with opportunities to tailor sessions according to the needs and abilities of the child (see Table 2 for an overview of the structure of sessions and Appendix S1 for a detailed description). TAs received a comprehensive teaching manual, a set of finely graded reading books, a pack of phonics resources, and a copy of Letters and Sounds (DfES, 2007) when they attended training.

**Table 2:** Content and structure of the reading and language strand intervention sessions

Reading		Language	
	
Activity	Time (min)	Activity	Time (min)
Read ‘easy’ book (>94% reading accuracy)	2–3	New word introduced with written, spoken and pictorial examples. One per session or in pairs (e.g. on/in)	5
Read ‘instructional’ level book (90–94% accuracy) while TA takes ‘running record’	5	Game using new word to reinforce learning in multiple contexts	5
Sight word learning and revision	2–3	Use new word in oral activities	5
Letter-knowledge (including digraphs), oral phonological awareness games and linking of letters and sounds	5	Use new word in guided writing	5
Introduce new book/shared reading of instructional book	5		

The Reading Strand was based on Reading Intervention which is a combined approach that teaches reading and phonics together (Hatcher, Hulme, & Ellis, 1994). The Language Strand aimed to teach new vocabulary and promote appropriate and accurate use of new words in expressive language (oral and written). Teaching was based on the multiple context approach (Beck, McKeown, & Kucan, 2002) making use of visual supports throughout the activities and using simple games to reinforce learning (e.g. matching, sorting). Target words, taught in themes, were selected from a set of parent-completed vocabulary checklists (Down Syndrome Education International, 2000) identifying words which many children were not yet using or did not yet understand and which would be useful additions to children’s vocabulary. (Further details of the intervention programme are given in the online supplementary material).

Two TAs from each school were invited to attend 2 days of training on the educational needs of children with DS. Specific intervention training was given 2 days shortly before the intervention began with a further day after 10 weeks of delivery. New TAs who joined the project part way through the intervention phases were trained in school. TAs were supported by regular telephone/email contact and observed at least once a term to assess fidelity of implementation and provide individualised feedback. Observations were also used to rate TAs according to their effectiveness in delivering the intervention using a scale of 1 (excellent) to 3 (poor). The average TA rating was 1.41 (0.64).

A questionnaire was used to assess children’s participation in classroom literacy activities both prior to and in addition to the intervention. Questionnaires were returned for 36 children (intervention group *N* = 24; waiting group *N* = 12). Prior to starting the intervention, children were involved in book reading (64%; including independent, guided and class reading), phonics instruction (28%), sight word learning (25%) and making and reading personal books (31%). Literacy instruction provided in addition to the intervention included book reading (81%), phonics (28%) and sight word learning (14%). Eleven per cent of respondents indicated that intervention was the only literacy input children received.

## Results

Table 3 shows the means and standard deviations for all measures for each group at *t*1, *t*2 and *t*3 (pre-intervention, after the first 20-week intervention period and after the second 20-week intervention period). Four children withdrew from the intervention part way through the study (see Figure 1) but we obtained follow-up measures and included their scores in our analyses. As expected given random allocation, the intervention and waiting control groups did not differ reliably on any measure at *t*1 (Cohen’s *d*’s ranged from 0.03 to 0.35).

**Table 3:** Means (*SD*) on all outcome measures at pre-intervention (*t*1), mid-intervention (*t*2), and post-intervention (*t*3) for intervention and waiting control groups

		Intervention group			
		
		Intervention		Waiting control	
			
Test (maximum score)		*M* (*SD*)	Range	*M* (*SD*)	Range
Single-word reading (79)	*t*1	5.86 (10.41)	0–46	6.88 (12.43)	0–52
	*t*2	10.50 (12.01)	0–52	8.92 (13.59)	0–56
	*t*3	14.86 (14.02)	0–55	13.36 (16.48)	0–64
Letter-sound knowledge (32)	*t*1	15.36 (8.13)	0–28	13.12 (9.27)	0–30
	*t*2	22.29 (7.28)	6–31	16.35 (9.42)	2–31
	*t*3	23.46 (8.02)	2–32	20.50 (7.46)	1–31
Phoneme blending (10)^a^	*t*1	5.00 (1.94)	0–10	4.85 (2.52)	0–10
	*t*2	6.25 (2.35)	2–10	4.88 (2.55)	0–10
	*t*3	6.43 (2.35)	2–10	5.73 (2.59)	0–10
Nonword reading (6)	*t*1	0.52 (1.25)	0–5	0.96 (1.61)	0–6
	*t*2	0.96 (1.48)	0–6	1.04 (1.90)	0–6
	*t*3	1.48 (1.87)	0–5	1.12 (1.79)	0–6
Phonetic spelling (92)	*t*1	4.89 (17.87)	0–92	12.35 (23.85)	0–92
	*t*2	11.00 (21.84)	0–92	17.00 (26.98)	0–92
	*t*3	20.00 (28.39)	0–89	25.72 (32.93)	0–89
Taught expressive vocabulary; weeks 1–20 (24)	*t*1	5.07 (3.51)	0–13	5.00 (3.59)	0–13
	*t*2	8.50 (4.07)	2–17	6.77 (3.84)	1–15
	*t*3	9.21 (4.29)	2–19	9.54 (5.05)	0–18
Taught receptive vocabulary; weeks 1–20 (24)	*t*1	12.04 (4.83)	3–22	11.92 (3.20)	5–18
	*t*2	15.50 (3.55)	7–21	14.04 (3.67)	7–22
	*t*3	16.07 (3.89)	7–23	15.58 (4.00)	6–21
Taught expressive vocabulary; weeks 21–40 (24)	*t*2	6.32 (3.13)	0–11	6.27 (3.42)	1–15
	*t*3	9.89 (4.06)	0–17	8.46 (4.13)	0–15
Taught receptive vocabulary; weeks 21–40 (24)	*t*2	16.11 (4.39)	8–23	14.19 (4.06)	5–22
	*t*3	16.68 (4.01)	7–23	16.62 (3.32)	9–23
Expressive vocabulary (170)	*t*1	29.64 (11.85)	8–59	27.69 (13.88)	8–71
	*t*2	34.00 (11.72)	13–67	32.00 (13.43)	12–74
	*t*3	37.39 (14.41)	10–68	36.38 (11.96)	14–69
Receptive vocabulary (170)	*t*1	35.61 (12.00)	11–61	35.23 (15.25)	12–67
	*t*2	38.79 (11.85)	20–68	38.27 (12.54)	15–64
	*t*3	44.25 (12.95)	15–74	42.42 (15.07)	16–72
Expressive grammar (37)	*t*1	5.86 (5.38)	0–23	4.80 (5.63)	0–28
	*t*2	8.29 (6.29)	0–26	6.04 (5.54)	0–23
	*t*3	7.93 (5.42)	0–21	8.12 (6.59)	0–27
Expressive information (40)	*t*1	13.84 (7.26)	0–32	11.79 (6.39)	0–27.50
	*t*2	16.63 (7.38)	3.00–37.50	14.77 (7.25)	3.50–32.50
	*t*3	18.01 (6.73)	2.00–31.50	18.75 (8.48)	4.00–34.50
No. of sessions attended (200)	*t*1–*t*3	137.46 (28.89)	72–183	75.28 (17.67)	17–92

a A test of *Sound Isolation* (Hulme et al., 2009) was also administered at *t*1 but discontinued due to marked floor effects (*M* = 0.81, *SD* = 1.63, max = 12; skewness = 1.95, *SE* = 0.33; kurtosis = 2.65, *SE* = 0.64).

### Intervention effects

**Figure 2:**
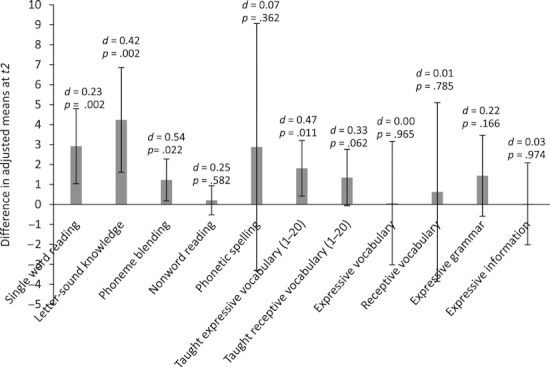
Comparison of the intervention and waiting control groups at *t*2 (controlling for *t*1), after receiving 20 and 0 weeks of intervention, respectively, on intervention outcome measures [with 95% confidence intervals, effect sizes (*d*, difference in raw score gains divided by pooled *SD* at *t*1)] and *p*-values

The effects of intervention on language and literacy outcomes were assessed using regression (ANCOVA) models implemented in Mplus (v 6.0; Muthén & Muthén 1998–2010). In these analyses the small amount of missing data was dealt with using Full Information Maximum Likelihood (FIML) estimators (the default in Mplus). To assess the impact of the intervention after the first 20 weeks, group differences at *t*2 were tested, controlling for baseline performance at *t*1, age and gender. The results are summarised in Figure 2, which plots the difference between the groups’ adjusted means (*t*2 scores controlling for covariates), with 95% confidence intervals. Any score greater than 0 represents greater progress in the intervention group compared to the waiting control group; where the 95% confidence intervals do not cross the *x*-axis, this represents a statistically significant effect (*p* < .05). The figure shows that children receiving intervention made significantly greater progress than those not receiving intervention on four primary outcome measures: single word reading, letter-sound knowledge, phoneme blending and taught expressive vocabulary (with small to medium effect sizes). The intervention effect did not transfer to other measures of literacy (spelling and nonword reading) or standardised tests of language (vocabulary, grammar and information).

**Figure 3:**
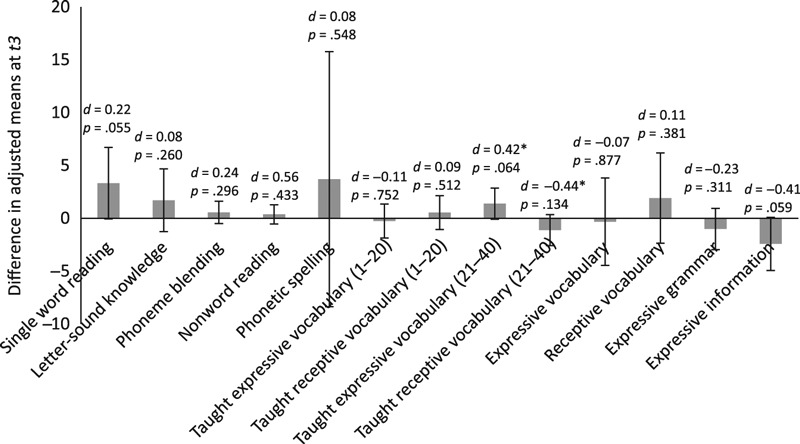
Comparison of the intervention and waiting control groups at *t*3 (controlling for *t*1 or *t*2*), after receiving 40 or 20 weeks of intervention, respectively, on intervention outcome measures [with 95% confidence intervals, effect sizes (*d*, difference in raw score gains divided by pooled *SD* at *t*1 or *t*2*)] and *p*-values

The data in Table 3 also indicate that once the waiting list control group began to receive intervention, their skills increased at about the same rate as those of the intervention group during their first 20-week period. To assess whether the intervention group remained ahead after the waiting control group had received 20 weeks of intervention, differences at t3 were tested, again controlling for baseline performance at *t*1 (except for taught vocabulary items introduced in the second block of intervention, where *t*2 scores are controlled), age and gender. The results are summarised in Figure 3. Although the children who had received 40 weeks of intervention were numerically ahead of those who received 20 weeks, none of the group differences were statistically reliable at this time point.

### Predictors of growth in reading

From the factors known to relate to response to reading intervention (after Nelson et al., 2003) we assessed behaviour, phonological awareness, letter knowledge, IQ, age and gender. We also assessed receptive language (after Vadasy et al., 2008 and Whiteley et al., 2007) – here the sum of *z*-scores for receptive grammar and vocabulary, which were highly correlated (*r* = 0.620); and extrinsic factors relating to length and quality of intervention. We derived a measure of reading growth across the 40-week period by computing the residualized *t*3 reading scores (controlling for *t*1 reading). Table 4 reports the correlations between these residualized reading gain scores, number of intervention sessions attended in the 40-week period, TA effectiveness rating, and key measures at *t*1. Growth in reading correlated with age (favouring younger children), TA effectiveness and attendance, whereas correlations with children’s rated behaviour problems and gender were not significant. Of the cognitive measures, letter knowledge was the only measure that correlated significantly with growth in reading. However, letter knowledge and receptive vocabulary correlated strongly with each other and receptive vocabulary also showed a sizable, but non-significant, correlation with growth in reading.

**Table 4:** Bivariate correlations between variables measured at *t*1 and progress in reading over 40 weeks (*t*3 controlling for *t*1), collapsed across groups

	1	2	3	4	5	6	7	8	9
1. Reading growth	–								
2. Age	−.34*	–							
3. Gender	.26	.09	–						
4. Behaviour	−.19	−.04	−.16	–					
5. Block design	.14	.36**	.02	−.28*	–				
6. Phoneme blending	.19	.29*	−.05	−.13	.35*	–			
7. Letter-knowledge	.36**	.11	.02	−.29*	.45***	.43**	–		
8. Receptive language	.23	.51***	.08	−.37**	.63***	.54***	.48***	–	
9. Sessions attended	.29*	.12	−.14	−.15	.32*	.10	.16	.14	
10. TA effectiveness^a^	−.29*	−.05	−0.18	0.30*	0.04	−0.12	−0.15	−0.22	−0.19

a Rating for second 20-week block of intervention for control group, and average across both 20-week blocks for intervention group – note that lower scores reflect higher effectiveness.

* *p* < .05;

** *p* < .01;

*** *p* < .001.

The relative strengths of all of these predictors were assessed in a multiple regression model predicting growth in reading (Table 5, Model 1). The model accounted for 51% of the variance in reading growth [*F*(9,42) = 4.81, *p* < .001]. Only age, receptive language and attendance were significant predictors in this model. These three predictors were entered into a second model (Table 5, Model 2), which accounted for 41% of variance in reading growth [*F*(3,48) = 11.22 *p* < .001]. All three variables accounted for independent variance, with age accounting for 28%, receptive language 19%, and attendance 9% of variance in reading growth. However, as noted above, letter knowledge and receptive language were highly correlated, and the absence of an effect of letter knowledge as a predictor in the models presented in Table 5 reflects this shared variance. If we substitute letter knowledge for receptive language as a predictor of reading progress in Model 2, letter knowledge is a highly significant predictor (B = 0.31; *t* = 3.15; *p* < .001) with the overall model (age, attendance, and letter knowledge) accounting for 35% of the variance in reading growth.

**Table 5:** Simultaneous multiple regression models predicting reading growth across 40 weeks from *t*1 measures

Predictor	*B*	*t*	*p*
Model 1			
Age	−0.56	−4.28	<.001
Gender	0.19	1.71	.095
Behaviour	0.10	0.81	.421
Block design	−0.05	−0.34	.734
Phoneme blending	0.07	0.49	.624
Letter-knowledge	0.20	1.54	.131
Receptive language	0.37	2.06	.045
Sessions attended	0.31	2.59	.013
TA effectiveness	−0.12	−0.96	.345
Model 2			
Age	−0.62	−4.82	<.001
Receptive language	0.51	3.97	<.001
Sessions attended	0.30	2.69	.001

TA, teaching assistant.

It should be noted that attendance was not manipulated in the trial (beyond the manipulation involved in assigning children to groups) and hence the effects of attendance on outcome need to be interpreted with some caution (it could be for example that the children least able to learn tended to show the poorest attendance).

## Discussion

This study is the first RCT of a reading and language intervention for children with DS. Children who received the intervention during the first 20 weeks of the trial made significantly more progress on several key measures (single word reading, letter-sound knowledge and phoneme blending; with small to moderate effect sizes) than children receiving their typical instruction. All of these skills were directly targeted by the intervention; little generalization was observed to reading-related abilities which rely heavily upon phonological skills, namely nonword reading and spelling. The children’s expressive knowledge of taught vocabulary had also improved. There were no gains in receptive vocabulary or on standardised tests of language (expressive and receptive vocabulary; expressive information and grammar).

The gains in single word reading were modest with an average gain of 4.5 words on the reading test per 20 weeks of intervention, compared to an average of two words when receiving typical literacy instruction. When interpreting these findings it is important to remember that these children have general learning difficulties. A similar intervention for DS by our group reported similar gains (two words per 8 weeks of phonics-based reading intervention), though with larger effect sizes (Goetz et al., 2008). That study pre-selected children with ‘emergent reading skills’, whereas no child in mainstream school was excluded from the present sample (indeed, some children had very poor oral language skills). Moreover, it needs to be noted that there was wide variation in the gains children made in reading, with some children making little or no progress and others making large and educationally significant gains. Importantly, 48/53 children were able to score on the reading test at *t*3 (compared with 32 at *t*1) and 10 children attained a standard score of 90 or above on the EWR reading measure at this time.

Another positive outcome of the present study was that improvements in reading continued for the group who received 40 weeks of instruction, and there was a dose-related relationship such that children who attended more sessions benefited more. Perhaps inevitably given a finite set of items to be taught, there was more limited growth in letter-sound knowledge during the second phase of intervention and gains in phoneme blending were also less good. However, it is not unusual for growth in word reading to be ahead of growth in phoneme awareness(Baylis & Snowling, 2012; Goetz et al., 2008) and even the modest improvement (*d* = 0.24) could be important in ensuring the future reading devleopment of these children (Stuebing, Barth, Cirino, Francis, & Flethcer, 2008).

The intervention had less impact on language outcomes though it was encouraging to find evidence of gains on the measure of directly taught expressive vocabulary (though not on the equivalent receptive vocabulary measure). Moreover, children maintained the gains they made in taught vocabulary beyond the initial teaching of those words when instruction moved to a different set of words. We hypothesize that this unexpected pattern of better gains in expressive than receptive knowledge for taught words is likely due to the emphasis placed upon using these words in different contexts during the sessions, as well as the differing task demands of the two tests. Nonetheless, the relatively poor progress of the group in oral language skills speaks to the fact that these children have pervasive language learning difficulties.

Once age and attendance were controlled, receptive language accounted for a significant proportion of variance in growth in reading, though, in contrast to findings from typical development (Muter, Hulme, Snowling, & Stevenson, 2004), phoneme awareness did not. In the present study, our measure of receptive language was a composite of vocabulary and grammar; statistically vocabulary had the stronger effect. Consistent with this, a longitudinal study of reading in DS (Hulme et al., 2012) also found that receptive vocabulary was a stronger predictor of reading skills than phoneme awareness. Taken together, these findings are consistent with the view that vocabulary knowledge places constraints on the development of segmental phonological representations in the absence of which, phoneme awareness is compromised (Metsala & Walley, 1998). Within the framework of the Triangle Model (Seidenberg & McClelland, 1989), the problems observed in the development of an efficient decoding system which can support nonword reading and phonetic spelling would be a natural consequence. Speculatively, our finding that younger children responded better to intervention may be explained by the fact that they were more likely to be exposed to phonics-based classroom literacy work which was reinforced by the current intervention but the optimal age for intervention in this population is an issue which deserves further research.

The intervention evaluated here was novel in its integrated approach to reading and language instruction for children with DS and is educationally realistic. Although the effect sizes obtained are modest and there was little evidence of transfer to broader measures of literacy or language the study does provide evidence to support the efficacy of the intervention. It is also worthy of note that the intervention was cost-effective since the TAs were already assigned to the children; furthermore, the control group could be considered conservative since all children worked with a dedicated TA delivering one-to-one instruction during the ‘waiting’ period. Response to the intervention was variable with children who were younger, received more intervention, and had better receptive language skills making better progress. A potential avenue for future research is to consider how best to tailor intervention to meet the needs of individual children.

## Supporting information

**Appendix S1** Summary of intervention programme (PDF document)
