# Supplementary material for: Efficacy of a reading and language intervention for children with Down syndrome: a randomized controlled trial
Source: J Child Psychol Psychiatry. 2012 Apr 26;53(10):1044–53. doi: 10.1111/j.1469-7610.2012.02557.x (PMC3470928; doi:10.1111/j.1469-7610.2012.02557.x)
Supplement: Supplementary file 1 [file jcpp0053-1044-SD1.pdf]

**Online supplementary material:** Burgoyne et al.: Efficacy of a reading and language intervention for children with Down syndrome: a randomized controlled trial

**Appendix S1:** Summary of intervention programme (PDF document)

*(See over)*

**Reading and Language Intervention for children with Down syndrome: Structure and content of intervention programme**

The intervention consisted of two interactive components: a Reading Strand and a Language Strand. These were delivered by trained teaching assistants (TAs) in daily 40-minute sessions to individual children in school. Sessions followed a 5-day cycle, with new content taught on days 1-4, and day 5 providing an opportunity to revise and consolidate new learning. Intervention content followed a prescribed programme but opportunities to tailor teaching to individual children's strengths and weaknesses were also incorporated. The teaching sessions were structured as outlined in Table 1 below.

Table 1: Session structure

| **Reading Strand (20 mins)** | |
|---|---|
| Reading easy level book (>94% accuracy) | 2-3 mins |
| Reading instructional level book (90%-94% accuracy) | 5 mins |
| Sight word learning | 2-3 mins |
| Letters, sounds, phonology | 5 mins |
| Introduce new instructional level book | 5 mins |
| **Language Strand (20 mins)** | |
| Vocabulary: Introduce new words | 5 mins |
| Vocabulary: Reinforce meaning of new words | 5 mins |
| Expressive language: Use new words in connected speech | 5 mins |
| Expressive language: Use new words in written language | 5 mins |

**Reading Strand**

The Reading Strand used a variety of books from a range of publishers which had been finely graded according to the Hatcher grading scheme. The books covered a range of topics and interests and were suitable for children at a range of reading ages from beginning readers to a reading age of 8 years. Three to four books were read at each level to enable consolidation before progressing to the next level of reading.

Specific learning targets and teaching activities used for sight word work and work on letters, sounds and phonology were chosen by TAs from suggestions provided during training and support sessions.

1. Easy book reading: The Reading Strand always started with reading an easy level book, that is, a book that the child can read with more than 94% reading accuracy. As the child progresses through the book levels, the number of books classified at the easy level increases. The child is given a choice of these books from which they select one to read to the TA at the start of the session.
2. Instructional book reading: Children then progress to reading a book which presents a slight challenge (can be read with 90-94% reading accuracy). This book will have been introduced by the TA in the previous session so that the child has some experience of it before reading it in this session. The child reads this book to the TA who takes a running record during reading to gauge the appropriateness of the book level and assess the child's reading behaviours. If

the child reads the book with >94% accuracy, a new instructional book is introduced at the end of the session. If the book is read with less than 94% accuracy, it is returned to at the end of session for the TA to read through with the child.

3. Sight word learning: A variety of games and activities are used to support sight word reading including word walls, word hunts, posting games, multi-sensory learning etc. Words to be learned by sight include high frequency and 'tricky' (irregular) words. A selection of words which have been learnt previously are revised every day alongside teaching of one or two new words (according to the child's own pace). A record of words children are learning and words they have previously learnt is maintained.
4. Letters, sounds and phonology: This section includes a) Learning of new letter sounds and digraphs in a structured sequential progression which maps on to phonics progression. This is done using similar games and activities used in the teaching of sight words e.g. flashcards and posting games, etc.; b) Listening to sounds where activities are directed at the rhyme and phoneme levels and make use of visual (pictures and objects) supports. This includes rhyme matching games, discriminating initial and end sounds (e.g. I-spy games, matching pictures according to beginning and end sounds, etc.) and blending and segmenting games using sound-talk; c) Linking letters and sounds for reading focusing on the phoneme level and using letters (magnetic/plastic/flashcards) for blending and segmenting activities. TAs were given suggestions for activities and were responsible for selecting their own targets and teaching tasks each day.
5. New instructional book reading: At the end of the session the TA read through an instructional level book with the child. This was a new book if the child had read the previous instructional book with >94% accuracy. In this case, the TA would go through the new book with the child, reading the story to them and discussing story content and pictures, etc. If the previous instructional book was read with less than 94% accuracy, this book was returned to at the end of the session and the TA would go through the book with the child, highlighting any words that the child found difficult.

**Language Strand**

The Language Strand worked on introducing and reinforcing the meaning of new words and on using the new vocabulary in expressive (spoken and written) language. Words taught in the programme were selected using parent-completed vocabulary checklists, targeting words which children did not yet have in receptive and/or expressive vocabularies and which would be useful in everyday contexts. These were grouped into themes (e.g. food, transport, school) to aid retention and foster semantic links. Each theme targeted a range of word types (nouns, verbs, adjectives and prepositions) which were taught over a fortnight. Typically, one new word was introduced each day; however, words were introduced in pairs where it made more sense to do so. A typical fortnight of teaching is presented in Table 2. Work was predominantly focused on the base form of the word with opportunities to extend this to look at, for example, verb tenses.

Daily records of language learning were kept in fortnightly themed topic books, which children played an active role in creating. These included written words that were taught, word webs and records of the teaching activities including personal pictures of children engaging with the words. Topic books were useful for revision and consolidation activities, and for keeping a record of the child's achievements which could be shared with parents and other staff in school.

Table 2: Fortnight of language teaching targets (food theme)

| Week | Monday | Tuesday | Wednesday | Thursday | Friday |
|---|---|---|---|---|---|
| 1 | Soup | Cereal | Chew | Lick | Consolidation |
| 2 | Crunchy | Soft | On | In | Consolidation |

Each teaching session was structured as follows.

1. Introduce new word: The TA introduced the new word to the child, providing spoken, written and visual representations of the new word in multiple contexts. The child was prompted to repeat the target word in imitation, supporting memory and articulation of the word. The TA then discussed the new word with the child, using an assortment of pictures (and, where possible, real objects) representing the word in a range of contexts. The child was also shown a flashcard of the word providing a written representation of the new vocabulary. The TA and the child then created a word web, linking the new word to the child's personal experience and understanding of the word meaning.
2. Playing a game using the new word: This was used to increase the depth of the child's understanding and experience of the new word by discussing the word meaning in multiple contexts. The child was given the opportunity to see, hear and say the word many times to promote a rich and multicontextual representation of the word meaning. Games were based on simple rule structures e.g. sorting games, Simon Says, rhymes and actions, matching games etc. and made good use of visual supports. Suggestions to enable TAs to make the games more or less challenging were provided for each game. It would be here that TAs could extend instruction beyond the base form of the word, if appropriate.
3. Developing expressive spoken language: Children were supported by TAs to generate a spoken utterance using the new word. This was guided by the child's level of ability and scaffolded by the TA. Pictures and games used in the session could be used to support the generation of spoken language and ensure this was meaningful and personally relevant to each child. TAs supported spoken attempts and extended children's utterances to model appropriate grammar.
4. Developing written language: Spoken words and sentences which were generated by the child and supported by the TA were recorded in written form in a themed topic book. Again, this was guided by the child's level of ability and scaffolded by the TA; activities included independent writing, tracing of key words and sentences, forming sentences using words on flashcards, etc. The emphasis was on the child's active involvement in creating topic books that had personal relevance.
